# Reduced susceptibility to pyrethroid insecticide treated nets by the malaria vector *Anopheles gambiae s.l*. in western Uganda

**DOI:** 10.1186/1475-2875-7-92

**Published:** 2008-05-26

**Authors:** Rubaihayo John, Tukesiga Ephraim, Abaasa Andrew

**Affiliations:** 1 Public Health Department, Mountains of the Moon University, P.O.Box 837, Fort-Portal, Uganda; 2 Vector Control Division, Medical Department, Kabarole District, Uganda; 3 Medical Research Council, Entebbe, Uganda

## Abstract

**Background:**

Pyrethroid insecticide-treated mosquito nets are massively being scaled-up for malaria prevention particularly in children under five years of age and pregnant mothers in sub-Saharan Africa. However, there is serious concern of the likely evolution of widespread pyrethroid resistance in the malaria vector *Anopheles gambiae s.l*. due to the extensive use of pyrethroid insecticide-treated mosquito nets. The purpose of this study was to ascertain the status of pyrethroid resistance in *An. gambiae s.l*. in western Uganda.

**Methods:**

Wild mosquitoes (1–2 days old) were exposed in 10 replicates to new nets impregnated with K-othrine (Deltamethrin 25 mg/m^2^), Solfac EW50 (Cyfluthrin 50 mg/m^2^) and Fendona 6SC (Cypermethrin 50 mg/m^2^) and observed under normal room temperature and humidity (Temperature 24.8°C–27.4°C, Humidity 65.9–45.7). A similar set of mosquitoes collected from the control area 80 km away were exposed to a deltamethrin 25 mg/m^2 ^impregnated net at the same time and under the same conditions. The 10-year mean KDT_50 _and mortality rates for each of the three pyrethroid insecticides were compared using the Student *t*-test.

**Results:**

A significant increase in the mean knockdown time (KDT_50_) and mean mortality rate were observed in almost all cases an indication of reduced susceptibility. The overall results showed a four-fold increase in the mean knockdown time (KDT_50_) and 1.5-fold decrease in mortality rate across the three pyrethroid insecticides. There was a significant difference in the 10-year mean KDT_50 _between deltamethrin and cyfluthrin; deltamethrin and cypermethrin, but no significant difference between cyfluthrin and cypermethrin. The 10-year mean difference in KDT50 for mosquitoes exposed to deltamethrin from the control site was significantly different from that of mosquitoes from the intervention site (p<0.05, t=3.979, 9df). The 10-year mean difference in mortality rate between deltamethrin (84.64%); cyfluthrin (74.18%); cypermethrin (72.19%) and the control (90.45%) showed a significant decline in mortality across all the three insecticides.

**Conclusion:**

Generally the results showed a trend of increase in mosquito resistance status with cross-resistance against all the three pyrethroid insecticides. This study reveals for the first time the development of pyrethroid resistance in *An. gambiae s.l*. in Western Uganda. It is therefore strongly recommended that the impact of this development on malaria control efforts be closely monitored and alternative fabric treatments be considered before this problem curtails community wide implementation of this malaria control strategy in Uganda.

## Background

In Africa, malaria is still the leading cause of childhood and maternal ill health and death [1]. In Uganda the burden of malaria is unacceptably very high being accountable for 20–40% of out-patient attendance at health facilities, 14% of in-patient deaths and 20–23% of childhood mortality[2]. The Ministry of Health in Uganda estimates 70,000–110,000 malaria-specific deaths every year and US$24.7 annual per capita expenditure on malaria treatment [2]. The problem has been compounded by the evolution of both drug and insecticide resistance to the commonly used antimalarials and insecticides respectively [3][4][5][6][7]. A number of novel technologies have been developed to combat malaria including insecticide treated nets (ITNs) and malaria vaccines but the vaccines have shown limited efficacy and need further development [3]. Pyrethroid insecticides being cost-effective, highly insecticidal at low dosage and highly biodegradable with low mammalian toxicity were recommended by WHO Pesticides Evaluation Scheme (WHO-PES) for large-scale use on mosquito bed nets for malaria prevention [4]. Trials of pyrethroid-impregnated mosquito bed nets demonstrated protective efficacy in the range of 20–70% with potential to alleviate the burden of malaria in sub-Saharan Africa [5-7]. Pyrethroid-impregnated nets are therefore being massively scaled-up in Africa, but there is serious concern about the likely evolution of widespread pyrethroid resistance. In Kenya, as early as 1991, there was a reduction in permethrin susceptibility in *Anopheles gambiae *one year after introduction of small-scale pyrethroid treated nets, but not from villages without the nets [8]. Pyrethroid resistance in *An. gambiae s.s*. has also been widely reported in West and Central Africa [9-13]. This resistance is mainly associated with target site insensitivity arising from a single point mutation in the sodium channel gene, often referred to as knockdown resistance (*kdr*) characterized by a leucine-phenylalanine mutation in West Africa [14] and leucine-serine mutation in East Africa [15]. Both West and East African *kdr *mutations have recently been reported in a population of *An. gambiae s.s*. from Central Africa [11,12]. In view of the above reports and the fact that no such a study had ever been conducted in Uganda, a cross-sectional survey was conducted in Kamwenge District of western Uganda, where donor-funded integrated malaria control programmes involving distribution of free pyrethroid -treated nets have been going on since 1998. The purpose of this study was to determine the prevalence of pyrethoid insecticide resistance in malaria vectors in this region.

## Materials and methods

Kamwenge District in western Uganda was purposefully selected because previous malariometric surveys showed that the district is holoendemic for malaria and as such it had benefited from the German Technical Cooperation(GTZ) funded Basic health malaria control programmes and many other malaria programmes under various NGOs funded by the Global Funds for HIV/AIDS, Malaria and TB. The design of the study was a multi-stage cross sectional survey with time taken for the median mosquito to be knocked down by each of the three pyrethroid insecticides and the 24 hrs post-exposure mortality rate being the units of observation once every year. Mosquito pupae were collected from roadside water pools from five parishes, namely Butemba, Kiziba, Rukooko, Masaka and Nkongoro, and raised to adults in the laboratory. All the mosquitoes collected were identified using morphological keys [16] and they all belonged to *An. gambiae *complex. One to two days old adult mosquitoes were exposed to new nets impregnated with K-othrine (Deltamethrin 25 mg/m^2^), Solfac EW50 (Cyfluthrin 50 mg/m^2^)and Fendona 6SC (Cypermethrin 50 mg/m^2^) and observed under normal room temperature and humidity (temperature 24.8°C–27.4°C, humidity 65.9–45.7) for the time taken for the median mosquito (50%) to be knocked down and for the 24 hrs post-exposure mortality rate. Each year, new nets were bought and cut into pieces and wrapped on locally made mosquito cages and thereafter a batch of eleven mosquitoes introduced into the cages in 10 test replicates for each of the three insecticides. A similar set of mosquitoes collected from the control area 80 km away were exposed to a deltamethrin 25mg/m2 impregnated net at the same time and under the same conditions. The control area was purposively selected, with hypo-endemic malaria, relatively cool and had no mosquito control bed net project because of low malaria prevalence. The bed net usage in the control area was estimated at < 5% while in the intervention area at 80% as malaria prevalence was very high (70–80% of OPD attendance). All knocked down mosquitoes were removed from the cages by use of a mouth aspirator, put in recovery cups provided with glucose soaked in cotton wool and mean mortality rate after 24 hrs recorded. The mean values for the KDT_50 _and mortality rates for the three pyrethroid insecticides were compared using the Student *t*-test. It was assumed that if 10 measurements with a sample of 110 mosquitoes per year are made for each insecticide with an alpha error of 0.05 and standard deviation of 8.0 (from previous studies), a comparison of the mean knockdown times for the three insecticides would give a power to detect a 8% increase in knockdown time per year of 80.43%. Therefore, a sample of 110 mosquitoes per insecticide per year was considered sufficient to detect the desired effect i.e. a significant decline in pyrethroid effect over time.

## Results

The susceptibility of adult mosquitoes (reared from larval collection) to deltamethrin, cyfluthrin and cypermethrin from 1998–2007 is presented in Table 1.

**Table 1:** Mean mortality rate and mean knockdown time (min) for the median (50%) in a batch of 11Anopheles mosquitoes in 10 replicates exposed to K-othrine (Deltamethrin 25 mg/m^2^), Solfac EW50 (Cyfluthrin 50 mg/m^2^)and Fendona 6SC (Cypermethrin 50 mg/m^2^) impregnated mosquito nets.

**Year**	**Deltamethrin**		**Cyfluthrin**		**Cypermethrin**		Control*	
	**KDT**_**50 **_**mortality**	**%**	**KDT**_**50 **_**mortality**	**%**	**KDT**_**50 **_**mortality**	**%**	**KDT**_**50 **_**mortality**	**%**
1998	19.2	100	21.7	90.2	20.3	88.7	19	100
1999	23.9	100	22.6	89.14	24.2	86	21.2	100
2000	25.4	100	28.1	82.7	27.6	78	20	100
2001	33.6	89.1	42.3	80.7	40.8	74.5	22	100
2002	38.5	90.2	51.5	77	52.8	72.5	23.2	80
2003	46.2	82	56.7	74	61.7	67.3	22	90
2004	50.8	76.1	62.9	66	67.4	69.5	21.4	89
2005	62.1	74	72.4	64.5	74.6	66.2	22.5	85.5
2006	65.9	69.8	77.3	61	83.2	60.8	25	80
2007	72.7	65.2	90.2	56.6	88.4	58.4	24.5	80

**Mean**	**43.83**	**84.64**	**52.57**	**74.18**	**54.10**	**72.19**	**22.08**	**90.45**

* Deltamethrin

The resistance status of the mosquitoes was based on the increase in the mean knockdown time(KDT_50_) and decrease in the mean mortality rates. The overall results showed a four-fold increase in the mean knockdown time (KDT_50_) and 1.5-fold decrease in mortality rate across the three cyano-pyrethroids from 1998–2007. The 10-year mean KDT50 for mosquitoes exposed to deltamethrin from the intervention site was 43.83(95%CI =30.37-57.28) which showed a significant increase over the 10-year period (p<0.05, t=7.4, 9df) while  that of cyfluthrin was 52.57(95%CI=35.5-69.6) and cypermethrin was 54.1(95%CI=36.2-71.9) both of which showed a significant increase over the 10 year period (p<0.05, t=6.9, 9df). The 10-year mean KDT50 for mosquitoes exposed to deltamethrin from the control site was 22.08(95%CI=20.75-23.41) which was significantly different from that of mosquitoes from the intervention site (p<0.05, t=3.979, 9df). The 10-year mean difference in mortality rate for mosquitoes in the intervention site exposed to deltamethrin 84.64% (95%CI = 75.25–94.03); cyfluthrin 74.18% (95%CI = 65.76–82.60); cypermethrin 72.19(95%CI = 65.08–79.30) and mosquitoes from the control site exposed to deltamethrin 90.45 (95% CI = 84.06–96.84) was significant (p < 0.05) and showed a significant decline in mortality across all the three pyrethroid insecticides.

## Discussion

Pyrethroid insecticides are neurotoxins and share many characteristics with DDT including a negative temperature coefficient, a rapid knockdown effect followed by a lethal effect [17]. Neurophysiological studies show that the knockdown effect is caused by poisoning of the peripheral nerves [18,19] and the lethal effect is due to an irreversible damage to both the peripheral and central neurons which occurs when poisoning takes long [20]. At the molecular level, this poisoning tend to interfere with the sodium channels, GABA (gamma-aminobutyric acid) activated chloride channels and membrane bound ATPase causing permanent depolarization of the peripheral and central neurons[18,19]. Preferential heavy use of pyrethroids has as earlier predicted resulted in extensive selection for resistance in many agricultural pests and various disease vectors [21,22]. Insecticide target site alteration conferred by the *kdr *gene and metabolic resistance due to amplification of oxidative detoxifying enzymes (acetylcholinesterases or glutathione-s-transferases) and co-factors, e.g. cytochrome P450 [17,23,24], have been widely reported in *An. gambiae s.l*. due to selection pressure as a result of extensive use of pyrethroid insecticides for malaria control and pest control in Agriculture [9-13,23]. However, most studies have shown that reduced susceptibility to pyethroid insecticides by *An. gambiae s.l*. is mainly due to increased target site insensitivity caused by the *kdr *allele [9,14,24,25]. Increase in the knockdown time as well as reduction in mortality rate have been accepted as an indicator of decline in the pyrethroid effect in *An. gambiae s.l*. [8,9,13,25]. A comparative analysis of the mean knockdown time (KDT_50_) and the mean post-exposure 24 hrs mortality rate for the three pyrethroid insecticides showed a significant increase in the mean KDT_50 _and a significant reduction in the mean mortality rate over the 10 year-period, an indication of the existence of knockdown resistance conferred by *kdr *alleles and/or metabolic resistance in *An. gambiae s.l*. in western Uganda. Although the 24 h post-exposure mortality rate using deltamethrin showed a slight decrease compared to cyfluthrin and cypermetrin, the overall reduction in the mean mortality rate across all the three cyano-pyrethroids over the 10 year period suggests cross-resistance to all pyrethroids. The slight decline in mortality rate and increase in KDT_50 _observed in the control mosquitoes could be explained by the possibility of *kdr *alleles as a result of preferential use of pyrethroid agro-chemicals in agriculture [21,25]. However, follow up studies need to investigate this possibility. This means that the fight against pyrethroid resistance needs concerted effort by all stakeholders and alternate use of pyrethroid insecticides with other types of insecticides in both public health and agricultural production is urgently needed in order to eliminate any differential advantage of the resistant individuals to pyrethroid insecticides.

There were some limitations in this study. First, Molecular differentiation of sibling species in *Anopheles gambiae* complex was not done which means that sibling species composition may differ between the intervention and control study sites. 

Secondly it was not possible to use a laboratory susceptible colony for the control test because it was not available anywhere in Uganda. This would be an ideal comparison as it would ensure consistent 100% mortality and a constant KDT_50 _in the control mosquito population.

## Conclusion and recommendations

Generally the results showed a trend of increase in mosquito resistance status with cross-resistance against all the three pyrethroid insecticides. This study reveals for the first time the development of pyrethroid resistance in *An. gambiae s.l*. in western Uganda. However, there is need for a molecular confirmation of the existence of the *kdr *alleles or to establish the type of resistance and extend the research to others areas in Uganda. At operational level, there is great need for continuous pyrethroid susceptibility testing in monitoring the efficacy of pyrethroid insecticide treated-mosquito nets. In addition, other alternative chemical compounds with little likelihood of cross resistance need to be developed before ITNs use in Uganda is curtailed by widespread pyrethroid insecticide resistance.

## Declaration of competing interests

The authors declare that they have no competing interests.

## Authors' contributions

RJ Developed the study design, participated in data collection, analysis and manuscript writing

TE Participated in data collection and data entry

AA Developed the data analysis plan, was responsible for data analysis and participated in manuscript writing

All authors read and approved the final manuscript.
